# Physical exercise in attention deficit hyperactivity disorder – evidence and implications for the treatment of borderline personality disorder

**DOI:** 10.1186/s40479-019-0115-2

**Published:** 2020-01-06

**Authors:** Aylin Mehren, Markus Reichert, David Coghill, Helge H. O. Müller, Niclas Braun, Alexandra Philipsen

**Affiliations:** 1 Department of Psychology, Biological Psychology Lab, European Medical School, University of Oldenburg, Oldenburg, Germany; 2 Department of Applied Psychology, Mental mHealth Lab, Institute of Sports and Sports Science, Karlsruhe Institute of Technology (KIT), Karlsruhe, Germany; 3 Department of Psychiatry and Psychotherapy, Central Institute of Mental Health, Medical Faculty Mannheim, Heidelberg University, Mannheim, Germany; 4 Royal Children’s Hospital, Melbourne, Victoria Australia; 5 Department of Psychiatry and Psychotherapy, University of Bonn, Bonn, Germany

**Keywords:** Exercise, Physical activity, Attention deficit hyperactivity disorder (ADHD), Borderline personality disorder (BPD), Cognitive, Executive function, Attention, Intervention, Treatment

## Abstract

A growing body of literature indicates a potential role for physical exercise in the treatment of attention deficit hyperactivity disorder (ADHD). Suggested effects include the reduction of ADHD core symptoms as well as improvements in executive functions. In the current review, we provide a short overview on the neurophysiological mechanisms assumed to underlie the beneficial effects of exercise. Further, we review the current evidence from experimental studies regarding both acute exercise and long-term interventions in ADHD. While the positive effects observed after acute aerobic exercise are promising, very few well-designed long-term intervention studies have been conducted yet. Moreover, although exercise effects have not yet been studied in borderline personality disorder (BPD), in the end of this paper we derive hypotheses why exercise could also be beneficial for this patient population.

## Background

Physical exercise is known to have positive effects on general health and well-being [123], to bear potential to improve mood and quality of life [48, 129], and to reduce stress responses [131]. In addition, a growing body of literature suggests beneficial effects of exercise on symptoms of attention deficit hyperactivity disorder (ADHD). Improvements in neurobehavioral functions have been demonstrated, including reduced impulsivity and hyperactivity, improved attention, and enhanced performance on executive functioning tasks [14, 16, 45, 82]. Moreover, an association between increased exercise levels and alleviated ADHD symptoms in the general population has been found [8]. Interestingly, the neurophysiological changes found to be induced by exercise considerably overlap with the neuropathological mechanisms implicated in ADHD [141].

In the following paper, we first provide a short overview on neurophysiological mechanisms suggested to underpin the beneficial effects of exercise on cognition and behavior. We then review results from studies with different experimental approaches (i.e., acute effects and long-term interventions) to investigate the effectiveness of exercise in improving ADHD symptoms. We complement previous reviews by providing a comprehensive overview on effects of different types of exercise on behavioral, cognitive, and neurophysiological parameters in ADHD, including the most recent studies in children, adolescents, and adult patients. Finally, we discuss overlapping symptoms and neurophysiological substrates of ADHD and borderline personality disorder (BPD). On this basis, we provide a first attempt to discuss potential benefits of exercise for BPD and encourage research endeavours.

### Neurophysiological effects induced by physical exercise

Neurophysiological effects of exercise include increased central arousal associated with elevated release of fronto-striatal neurotransmitters such as dopamine, epinephrine, norepinephrine, and serotonin [4, 18, 81, 84]. In ADHD patients, abnormalities in fronto-striatal functioning, in particular hypoactivity in the dopaminergic and noradrenergic systems, have been related to the attentional and executive impairments [12, 30, 103, 107, 134]. Stimulants are the first-line medication in ADHD [103, 104]. These extremely effective medications increase the availability of dopamine and norepinephrine in the prefrontal cortex and result in a reduction of symptoms and improvement of executive functioning in the majority of patients [22, 25, 87, 113, 118, 133]. In a similar way, exercise might compensate for dysregulated catecholamine levels in ADHD and thereby improve cognitive and behavioral functioning [141].

Similarly, diverse studies have suggested that dysregulations in fronto-striatal neurotransmitter systems might contribute to the development of BPD. In particular, genetic variations in the serotonin system have been shown, but also alterations in dopaminergic and noradrenergic functioning as well as in the endogenous opioid system have been proposed [6, 13, 36, 92, 95, 138, 146]. Notably, besides increased levels of catecholamines and serotonin, also endorphins are released during exercise [38, 85], which might not only enhance or stabilize mood and contribute to reward experiences during prolonged exercising, but might also modulate emotional functioning and stress reactivity, which are core hallmarks of BPD [67, 91]. Further proposed mechanisms of action include an upregulation of growth factors, such as the brain-derived neurotrophic factor (BDNF) [33, 69]. BDNF is expressed in the hippocampus and plays a crucial role in brain development and plasticity as well as in learning and memory processes [64, 71]. Interestingly, dysregulation of BDNF has also been implicated in ADHD [70, 130] and in BPD [89, 100].

Experimental studies on the neurophysiological effects of exercise have mainly been conducted in animals. In rodents, changes in central neurotransmitters and neurotrophins have been demonstrated consistently after single bouts of exercise as well as after longer phases of regular exercises [27, 85, 135]. In animal models for ADHD, exercise-induced catecholamine and BDNF increases have been related to cognitive enhancements (e.g., [54, 59, 112]). While these results from animal studies are fairly robust, findings in humans are more heterogeneous and depend on more specific exercise characteristics, such as duration and intensity (e.g., [127, 149]). In addition, only peripheral concentrations of neurotransmitters (i.e., plasma or serum) have been measured in humans so far, and these may not accurately reflect central concentrations. Very few studies have investigated exercise-related changes in brain metabolism using positron emission tomography (PET) and these studies have themselves revealed mixed results. While Boecker et al. [9] found evidence for reductions in opioid receptor availability after a single session of exercise, Wang et al. [140] did not detect any changes in dopamine receptor availability. Neuroimaging and electrophysiological studies have, however, demonstrated changes in brain structure and function after exercise interventions in several participant groups, supporting the proposed neuroprotective effects of exercise. These changes include gray matter increases in frontal [24] and hippocampal areas [33, 97], as well as modified brain activation patterns and changes in functional connectivity [60, 137]. In both ADHD and BPD, structural and functional abnormalities in fronto-striato-limbic brain areas have been demonstrated [26, 62, 120, 121]. ADHD and BPD often co-occur within individuals [101], share common genetic factors, and co-aggregate in relatives [35, 63, 77]. Consequently, it seems probable that those patient groups could benefit from physical exercise.

However, so far only very few studies have assessed the effects of exercise on neurophysiological parameters in patients with ADHD, and for BPD there are no studies upon this issue at all. Wigal et al. [142] tested the effects of a single session of cycling on plasma catecholamine levels in boys with ADHD and without ADHD. For both groups, they found epinephrine and norepinephrine increases after exercise. The increases were, however, smaller in the ADHD group than in the healthy control group. Moreover, dopamine levels only increased in healthy participants, but not in those with ADHD. In contrast, in a group of children with ADHD, Tantillo et al. [128] observed acute exercise-related changes in spontaneous eye blink rate and acoustic eye blink startle response, which are considered as noninvasive indicators of dopaminergic activity as they are sensitive to dopamine agonists. These results were, however, dependent on exercise intensity and differed between boys and girls. Changes in boys were only observed after maximal exercise, while changes in girls were only observed after submaximal exercise. Gapin et al. [39] focused on acute exercise-related BDNF serum changes, but could neither find any changes in young adults with ADHD nor in healthy controls. Finally, one study assessed peripheral epinephrine and serotonin levels after a long-term exercise intervention in a small group of boys with ADHD [65]. After 3 months of mixed exercises, the boys showed significant increases in epinephrine concentration, whereas serotonin levels did not increase significantly. To conclude, evidence from empirical studies supporting the suggested mechanisms of exercise in patients with ADHD is still scarce and neurophysiological changes due to exercise need to be further investigated. In addition, up to now, no research has linked the neurophysiological effects of exercise to BPD.

### Neurocognitive effects of physical exercise in ADHD

#### Acute effects

There is increasing evidence that a single session of exercise can lead to immediate improvements in ADHD symptoms and cognitive functions. Studies have mainly focused on the effects of aerobic exercise (e.g., cycling or running) with moderate intensities and minimum durations of 20 min on executive and attentional functions.

In children with ADHD, the majority of studies have revealed positive effects of acute exercise on executive task performance with small to large effect sizes. Most studies compared task performance after exercise to task performance after a cognitively and physically undemanding control condition (e.g., watching a video). The studies have hereby identified exercise-induced improvements in response inhibition, impulsivity, and attention as assessed by the flanker task [72, 105], Go/No-go task [21], and Stroop task [17, 102]. In addition, exercise-induced enhancements in task switching [17, 50,] and cognitive flexibility [73] have been shown. Piepmeier et al. [102] further demonstrated that some aspects of executive functioning seem to benefit from acute exercise while others do not. Compared to watching a movie, both children with ADHD and healthy controls showed faster reaction times in all conditions of the Stroop Task after 30 min of cycling. However, exercise did not improve performance on the Tower of London and the Trail Making Test, which are measures to assess planning and problem solving as well as cognitive flexibility and set-shifting. Another study found benefits in academic performance following exercise [105]. Both children with ADHD and healthy controls improved in reading comprehension and arithmetic after 20 min of moderate aerobic exercise compared to a seated reading condition.

The number of studies complementing behavioral outcomes with electrophysiological measures has increased over the last years. To our knowledge, Pontifex et al. [105] were the first to explore the electrophysiological effects of acute exercise in ADHD using electroencephalography (EEG). They observed an amplitude increase and latency decrease in the P300 component during a flanker task following acute exercise. The P300 component is an important electrophysiological subcomponent within the event-related potential that is typically associated with the allocation of attentional resources [104]. These findings were independently replicated in 2017. Using a similar flanker task paradigm, Ludyga et al. [72] also observed exercise-induced P300 amplitude increases in children with ADHD as well as in healthy children. Hung et al. [50] further demonstrated exercise-related P300 amplitude increases during a task switching paradigm. All three studies in addition observed improvements in behavioral task performance, supporting enhanced attention and inhibition due to exercise. Chuang et al. [21] investigated the effects of acute exercise on the contingent negative variation (CNV), another component of the event-related potential. The CNV is regarded as an electrophysiological marker for anticipatory attention to an upcoming stimulus and preparation of motor response [10, 139]. In this study, a group of children with ADHD performed a Go/No-go task after 30 min of treadmill running and after a control condition. After exercise, they showed shorter reaction times and a decreased CNV amplitude, which the authors interpreted as facilitation of motor preparation.

Far fewer studies have been conducted with adults with ADHD. Gapin et al. [39] assessed the effects of 40 min of moderate exercise on different aspects of executive function in 10 young adults with ADHD and 10 healthy controls. While healthy controls showed exercise-related improvements in all cognitive domains assessed, ADHD patients only improved in a response inhibition task, but not in working memory or task switching. It should be noted, however, that in this study the authors did not compare task performance after exercise to a control condition. In another adult ADHD study, Fritz and O’Connor [37] reported improvements in mood, motivation, fatigue, and depression after 20 min of cycling as compared to a control condition. Interestingly, no changes in vigilance or hyperactivity were observed in this study. A very recent fMRI study from our lab [86] investigated the effects of a single session of aerobic exercise on attention and executive function as measured by a flanker task in adult patients with ADHD and healthy controls. Following 30 min of cycling at moderate intensity compared to watching a movie, we observed improved reaction times in patients with ADHD but not in healthy controls. However, unlike as in the previously described EEG studies, no exercise-induced changes in brain activation were identified in this study. In a further exploratory analysis, however, for which the sample was divided into two groups according to individual fitness level, brain activation changes were found in task-related brain areas for those patients with a higher degree of cardiorespiratory fitness. This finding suggests a moderating role of fitness in acute exercise effects.

A few studies have also investigated the effects of different types of exercise, different intensities, and the role of medication status. Ludyga et al. [72], for instance, compared acute cognitive effects of aerobic exercise to those of coordinative exercise in children with ADHD. Coordinative exercise consisted of exercises requiring object control skills and bilateral coordination, while aerobic exercise included cycling at moderate intensity. Compared to a control condition (watching a video), both exercise types led to an improved flanker task performance and P300 amplitude increase, but the effects were larger after aerobic exercise. Two further studies examined the effects of intermittent high-intensity exercise and revealed mixed results. While Medina et al. [83] reported exercise-induced improvements in attention, Mahon et al. [75] did not find any enhancements. Noteworthy, results of studies examining the effects of acute high-intensity exercise in other participant groups are also highly heterogeneous [82]. Further, it is noteworthy that these two studies were the only ones which accounted for the role of medication status. While the beneficial effects observed by Medina et al. [83] were independent of medication status, Mahon et al. [75] reported even worsened performance after exercise when children were on medication.

To sum up, in line with findings in healthy participants and other clinical populations, beneficial effects of acute exercise on ADHD symptomatology are so far most robustly observed after aerobic exercise at moderate intensity. It should be noted, however, that studies on other exercise modalities and intensities are still very scarce and need to be further investigated. Also, patient characteristics like cardiorespiratory fitness and medication status should be more carefully considered when evaluating exercise effects.

#### Long-term interventions

Also the findings from long-term exercise intervention studies point towards positive effects on ADHD symptoms and related cognitive impairments. In most of these studies, patients participated in programs consisting of various cardio exercises over several weeks (e.g., running, swimming, cycling, rope skipping, ball sports, or sports games). Almost all studies found exercise-related improvements with small to large effect sizes in ADHD symptoms (inattention, hyperactivity, and impulsivity), executive functions, academic performance, or motor skills (for previous reviews see e.g., [14, 93]). However, most of these studies had methodological shortcomings, such as small sample sizes, no randomization or blinding procedure, no adequate control condition, or no healthy control group, and should therefore be interpreted with caution. Further, studies were highly heterogeneous with respect to patient characteristics (e.g., age, gender, diagnosis criteria, and medication status), exercise characteristics, and neurocognitive assessment. This heterogeneity as well as methodological considerations render it difficult to compare and to generalize the results.

As stated, some studies reporting beneficial effects of exercise interventions did not include any control condition (e.g., [44, 47, 124]) and thus may not rule out the possibility of practice effects. On the other hand, many studies compared an exercise intervention to a control group receiving no treatment and found positive effects on neurobehavioral performance, such as attention, working memory, inhibition, impulsivity, emotional functioning, motor skills, cognitive flexibility, or classroom behaviour [1, 15, 80, 88, 98, 99, 136]. At a first glance, these results seem promising, but the lack of an alternative treatment as control condition makes it almost impossible to separate exercise-specific effects from other factors such as increased patient care, participation in an intervention in general, and social engagement. In fact, results from the few available randomized controlled trials which included an active control condition or compared the effects of different exercise programs are by far more heterogeneous.

The crucial role of an active control condition is further underlined by a study by Bustamante et al. [11]. In this study, 35 children with ADHD and/or disruptive behavior disorders were divided into two groups, both of which participated in a 10-week after-school program on 5 days per week. The program consisted of various activities, which were identical for both groups, with the exception of one group-specific treatment hour. During that hour the intervention group participated in physically active games and exercises, whereas the control group took part in physically inactive games and arts. For both groups, the authors reported improvements in behavioral and neuropsychological outcome measures from pre- to post-intervention assessments. From this, they concluded that routines, engagement in activities, and behavior management strategies might facilitate ADHD symptomatology. In another study, Hoza et al. [49] demonstrated beneficial effects of 30 min before-school exercises on parent and teacher ratings of ADHD symptoms, moodiness, and peer functioning, which were for most measures greater than in the control group performing arts in a sedentary classroom setting. However, improvements in some of the assessed areas (e.g., peer functioning and teacher ratings in ADHD symptom severity) did not differ between the groups.

To investigate whether effects depend on the type of exercise, Ziereis and Jansen [148] divided 43 children with ADHD into two intervention groups and one non-active control group. The two intervention groups participated in a 12-week training program (one 60-min session per week) with different foci: While group 1 was trained in specific abilities like ball handling, balance, and manual dexterity, group 2 engaged in a non-specific exercise program that consisted of swimming, running, climbing, and sports games. The control group received no treatment at all. Both intervention groups, but not the control group, improved in working memory and motor performance, indicating that specific as well as unspecific exercises can have beneficial effects. However, also in this study a potential influence of other psychosocial factors such as an increased patient care cannot be fully ruled out.

Two randomized controlled trials investigated the effects of exercise in addition to pharmacological treatment. Kang et al. [57] divided 28 boys with ADHD into two groups, which both received methylphenidate and in addition a 6-week therapy program. While the exercise intervention group performed a 90-min workout twice a week that entailed various sports elements (running, throwing, rope skipping), the control group received 12 educational sessions for behavioral control. Both groups showed improvements in ADHD symptoms (attention, hyperactivity, impulsivity), executive functioning (trail making test), and social behavior. For most measures, these improvements were significantly greater in the exercise intervention group than in the control group. In addition, Choi et al. [19] included fMRI to investigate the effects of a 6-week adjunct exercise program in addition to methylphenidate treatment on Wisconsin Card Sorting test performance in adolescents with ADHD. They found that exercise in addition to medication improved task performance and increased frontal lobe activity to a greater extent than educational sessions and medication. Hence, both studies indicate that exercise can enhance the effects of medication.

In a large multi-centre randomized controlled trial that included 112 children with ADHD, the effects of neurofeedback on several outcome measures (e.g., ratings of ADHD symptoms, neurocognitive functions, EEG components) were compared to pharmacological treatment with methylphenidate and to physical exercise [41–43, 51, 52]. Methylphenidate was superior to neurofeedback and exercise in improving most outcome measures, while exercise had positive effects on only very few measures. It should be mentioned, however, that the study specifically focused on the effects of neurofeedback, while exercise was only used as control condition and differed from most exercise intervention studies in terms of frequency and intensity (30 sessions of 20 min moderate to vigorous intensity exercises over 10–12 weeks).

A few studies have also investigated the effects of yoga in ADHD patients, with heterogeneous results. Jensen and Kenny [53], for instance, divided 19 boys with ADHD, who were stabilized on medication, into an intervention group taking part in 20 weekly 1-h yoga sessions and a control group performing cooperative group activities once a month. Subjective measures (parent ratings) indicated some improvements in ADHD symptoms in both groups, whereas no improvements in a neuropsychological attention task were found. These findings indicate only limited effects of yoga and in addition no superiority of yoga compared to cooperative activities. Haffner et al. [46] compared the effects of yoga to those of a motor training. In a crossover design, 21 children with ADHD participated in two interventions, each consisting of 8 weeks of training that took place twice a week. The yoga intervention consisted of typical yoga poses and breathing exercises, while the motor training involved sports games such as tossing, catching, dexterity games, concentration, and group games. Both interventions had positive effects on attention and ADHD symptoms, but the effects of the yoga intervention were greater compared to the motor training. In line with this finding, Chou and Huang [20] also reported improvements in sustained attention and discrimination after 8 weeks of a yoga exercise program in 24 children with ADHD as compared to a control group receiving no intervention.

In conclusion, results from exercise intervention studies appear promising for developing alternative or adjunct treatment approaches for ADHD. At present, however, randomized controlled trials that included an active control condition do not reveal a clear superiority of exercise compared to other activities. Furthermore, many studies applied combinations of different exercise types, making it difficult to determine which elements actually caused the desired therapeutic effect. It will be a challenge of future studies to detect specific aspects of exercise interventions that lead to positive effects as well as to include adequate control conditions. In addition, including healthy control groups might provide insight if benefits due to exercise are more pronounced or specific to ADHD or might occur in diverse participant groups.

### Implications for BPD

Core symptoms of BPD include emotional dysregulation, impulsivity, identity disturbances, stress-related dissociation, non-suicidal self-injury, and suicidal behavior [3]. In the following, we illustrate how exercise affects selected BPD-relevant symptoms, and from this, we cautiously deduce conceivable effects of exercise in BPD which, of course, will have to be investigated by studies in BPD samples to derive substantial evidence. Here, we first highlight similarities between ADHD and BPD and try to apply the findings described in ADHD to BPD. Further, we incorporate relevant research conducted in different participant groups, which revealed enhancements in functions that are typically impaired in BPD.

To start with, there is a substantial overlap in the clinical presentation of ADHD and BPD (for a review, see [77]) and both disorders often co-occur [35, 101]. Both, ADHD and BPD are characterized by the clinical symptoms affective instability and impulsive behavior [77] as well as impairments in executive functioning [79, 114, 132]. The effects of exercise on executive functions and impulsivity have not only been demonstrated in ADHD patients, but also studies in healthy and diverse clinical populations have established robust evidence that those functions greatly benefit from engagement in physical exercise [16, 23, 82].

These benficial effects might be related to the fact that exercise-induced changes in neurophysiological processes mainly involve fronto-striatal brain functioning, which is highly relevant for cognitive and behavioral control [2, 5, 110, 111]. Notably, neuroimaging studies have found neurochemical, structural, and functional abnormalities in the prefrontal cortex in BPD patients [62] that overlap with brain changes in ADHD [120]. Similar to as in ADHD, exercise-related release of catecholamines could be a potential mechanism of action in BPD, not only improving executive functioning and reducing impulsivity, but also influencing mood-related symptoms. Likewise, structural and functional changes in prefrontal brain areas due to exercise [24] might positively impact BPD symptomatology.

In addition, limbic structures including hippocampus and amygdala have been identified as candidate endophenotypes for BPD [28, 62, 115]. Reduced hippocampal volume has been associated with behavioral symptoms like impulsivity [119]. Interestingly, there is increasing evidence that exercise and physical fitness are related with greater hippocampal volume in older adults [32, 33], possibly due to a prevention of age-related deterioration [34]. Furthermore, alterations in the endogenous opioid system and neuroendocrine responses mediated by the hypothalamic-pituitary-adrenal axis (e.g., cortisol) have been proposed in BPD, which may contribute to symptoms like emotional dysregulation and increased sensitivity to stress [6, 56, 125, 144, 145]. Exercise has shown to affect the endogenous opioid system [9] and to enhance mental stress sensitivity [7, 116, 131] and therefore might positively impact those symptoms in BPD as well.

Exercise-induced release of endorphins and catecholamines may also lead to enhancements in mood-related symptoms such as emotional dysregulation, affective instability, low mood, inner emptiness, or hopelessness. The therapeutic effects of exercise on these symptoms are well-known from studies in healthy participants and in patients with affective disorders (for recent reviews, see [55, 126]). Naturalistic studies have further shown ecological validity of those findings, providing evidence that physical activity and exercise behavior indeed relate to emotional (in) stability and the ability to regulate emotions in healthy populations [76, 108]. In a recently published study, Ligeza et al. [68] found that women who exercised on a regular basis showed more efficient control of negative emotions. One relevant study in this context [31] compared autonomic nervous system processes between patients with BPD and healthy controls. It was found that elevations of heart rate in BPD patients at rest and in response to emotional stimuli were fully mediated by exercise activities in the past year (less in BPD patients than in healthy controls). In another study from Dunton et al. [29], active children showed higher emotional stability compared to their less active counterparts. Moreover, a recent study across 661 participants aged 8–73 years found that people with higher fluctuations in perceived subjective energy showed less physical activity. From this, the authors concluded that instability in emotional states may either deplete self-regulatory capabilities for physical activity planning or that physical activity can stabilize emotional states [74]. Another common symptom in BPD is unbearable inner tension, which patients sometimes can only manage by engaging in non-suicidal self-injury, substance abuse, or other ultimately maladaptive behaviors. Interestingly, naturalistic investigations in community-based populations point towards a critical potential of physical activity to regulate affective states including inner tension [40, 58, 61, 66]. In particular, we [109] assessed physical activity via accelerometry and psychological states for 1 week in 106 adults during their daily routines and participants reported about their exercise activities. We found that exercise (i.e., structured activities characterized by high energy consumption such as jogging, skating, swimming, or playing tennis) and non-exercise activity (i.e., unstructured activities in everyday life such as climbing stairs to fetch papers from the basement) differed regarding their psychological effects. That is, while non-exercise activity increased energetic arousal and inner tension, exercise increased valence and calmness. Therefore, we propose that patients suffering from inner tension, such as BPD patients, may gain if they engage in exercise sessions.

An additional argument for the application of physical exercise in BPD patients are the well-known beneficial effects on further psychological and organic symptoms that may be of relevance in BPD. Among other areas, therapeutic effects have been demonstrated against obesity [143], cardiovascular disease risk and symptoms [96], body dissatisfaction [94, 106], and poor self-esteem [90, 147]. While these symptoms do not represent the core symptomatology of BPD, they often accompany a BPD, and physical exercise may help to alleviate these accompanying symptoms. In addition, exercise can increase self-efficacy [78], which may improve adherence to behavioral therapies [122].

In sum, there are several indirect empirical indications that physical exercise could be an interesting and helpful adjunctive intervention option for BPD. We therefore encourage empirical studies to explore the therapeutic potential of physical exercise in BPD patients.

## Conclusions

Results from experimental studies indicate potential benefits of both acute exercise and long-term exercise interventions for patients with ADHD. However, while fairly robust effects of acute aerobic exercise with moderate intensity on ADHD symptoms and executive functions have been demonstrated in children with ADHD, other exercise modalities and intensities as well as effects in adults have not been investigated sufficiently. Due to methodological shortcomings (e.g., lack of an adequate control condition), findings from long-term intervention studies have to be interpreted with caution. Nevertheless, the existing findings motivate further well-designed randomized controlled trials examining exercise as an adjunct or stand-alone therapy for ADHD.

Interestingly, in contrast to a broad range of studies on psychological interventions, exercise effects in BPD have not been researched yet. One reason for this could be that BPD is highly associated with altered body image and shame-proneness making it difficult to engage in physical activities [85]. However, due to partly overlapping symptoms, neuropathological correlates, and high comorbidity between ADHD and BPD and the beneficial effects evidenced in healthy and diverse clinical populations, we suggest to investigate whether exercise entails potential benefits for the therapy of BPD. Further advantages of physical exercise include low costs, easy implementation, absence of side-effects, an active role of the patient including possibly enhanced compliance, non-invasiveness, as well as additional psychological and physiological benefits.

## Data Availability

Not applicable.

## Abbreviations

ADHD: Attention deficit hyperactivity disorder
BDNF: Brain-derived neurotrophic factor
BPD: Borderline personality disorder
CNV: Contingent negative variation
EEG: Electroencephalography
fMRI: Functional magnetic resonance imaging
PET: Positron emission tomography
